# Mutation spectrum of Chinese patients with Bartter syndrome

**DOI:** 10.18632/oncotarget.21355

**Published:** 2017-09-27

**Authors:** Yue Han, Yi Lin, Qing Sun, Shujuan Wang, Yanxia Gao, Leping Shao

**Affiliations:** ^1^ Central Laboratory, Affiliated Hospital, Qingdao University, Qingdao 266003, P.R. China; ^2^ Department of Nephrology, Affiliated Hospital, Qingdao University, Qingdao 266003, P.R. China; ^3^ Pediatrics, Affiliated Hospital, Qingdao University, Qingdao 266003, P.R. China; ^4^ Department of Nephrology, Qingdao Women and Children's Hospital, Qingdao University, Qingdao 266011, P.R. China; ^5^ Department of Nephrology, Qingdao Branch of Qilu Hospital of Shandong University, Qingdao, Shandong 266000, P.R. China

**Keywords:** bartter syndrome, CLCNKB gene, SLC12A1 gene, BSND gene, genotype and phenotype

## Abstract

**Objective:**

Bartter syndrome (BS) has been rarely reported in Chinese population except for a few case reports. This investigation was aimed to analyze the mutations of the causal genes in sixteen Chinese patients with BS, and review their followup and treatment.

**Methods:**

Identify mutations by the next generation sequencing and the multiplex ligation-dependent probe amplification (MLPA). Clinical characteristics and biochemical findings at the first presentation as well as follow-up were reviewed.

**Results:**

15 different CLCNKB gene mutations were identified in fourteen patients with BS, including 11 novel ones. A novel missense mutation and a novel small deletion were found from SLC12A1 gene. A novel gross deletion was found in CLCNKA gene. A recurrent missense mutation was identified from BSND gene. We found that the whole gene deletion mutation of CLCNKB gene was the most frequent mutation (32%), and the rate of gross deletion was up to 50 percent in this group of Chinese patients.

**Conclusion:**

The present study has found 19 mutations, including 14 novel ones, which would enrich the human gene mutation database (HGMD) and provide valuable references to the genetic counseling and diagnosis of the Chinese population.

## INTRODUCTION

Bartter syndrome (BS) is a heterogenic autosomal recessive disorder of salt reabsorption at the thick ascending limb (TAL) of the loop of Henle, presenting as hypokalemic metabolic alkalosis with normotensive hyperreninemia and hyperaldosteronism [1–2]. According to the time of onset and characteristic symptoms, BS is clinically classified into antenatal BS (aBS) and classic BS (cBS). BS can also be divided into five subtypes based on the underlying mutant gene: SLC12A1 (600839, type I), KCNJ1 (600359, type II), CLCNKB (602023, type III), BSND (606412, type IVa), CLCNKB and CLCNKA (602024) co-mutated (type IVb), and CASR (601199, type V) [3]. More recently, a novel transient form of antenatal BS has been found to be caused by MAGE-D2 mutations [4].

So far, more than 100 Bartter Syndrome-related gene mutations have been reported [2]. However, BS has been rarely reported in Chinese population except for a few case reports [5]. This study has recruited the largest group of BS patients in China, then identified their mutations of causal genes, then analyzed their relationship between genotype and phenotype, as well as reviewed their treatment and follow-up, hoping to explore the mutation spectrum of Chinese patients with BS, and improve the clinician awareness of the disease in China.

## RESULTS

### Clinical manifestation and laboratory examination

#### Clinical signs and symptoms

16 unrelated Chinese patients from 12 different provinces were enrolled in this study (Table 1). Except for the polyhydramnios and premature delivery of patient B15 and C16, 10 patients were firstly admitted in 3-6 months after birth due to a fever caused by nonspecific sites of infections (10/14), 3 were due to growth delay after 7-month-old (3/14), while 1 patient admitted to our hospital at 3-month-old because of facial neuritis (1/14).

**Table 1:** The basic information and laboratory results of the sixteen patients with Bartter syndrome at the first admission

patient	Province	gen der	Age^a^	Age^b^	BP (mmHg)	laboratory results												
						Blood PH	CO_2_C P(mm ol/L)	SNa (mm ol/L)	SK (mm ol/L)	SCl (mmol/ L)	SMg (mmol/ L)	ALD(ng/L)	PRA (ng·ml-1·h-1)	UK/UCr (mmol/mmol) (<1.5)	UMg/UCr (mmol/mmol)	UCa /UCr (mm ol/mmol)	UCr (mmol/L)	eGFR(mL·min-1·1.73m2^-1^)
						(7.35- 7.45)	(22-28)	(135- 145)	(3.5- 5.5)	(99- 110)	(0.73- 1.06)	(12.0-157.5)	(0.05-0.79)					
A1	Zhejiang	M	3 mos	1yrs	59/34	7.48	29.9	133	2.44	95.5	1.06	308.9	7.70	47.6	1.20 (0.4-2.2)	0.67 (0.09-2.2)	18.2 (14-30)	119.5(30-86)
A2	Anhui	M	6 mos	1 yrs	65/45	7.63	42.5	127	1.84	78.3	0.81	294.0	11.60	73.7	1.52 (0.4-2.2)	0.50 (0.09-2.2)	20.3 (14-30)	119.6(39-114)
A3	Sichuan	F	4 mos	2 yrs	54/29	7.92	48.3	124	1.66	67.4	0.57	65.5	1.76	68.3	2.20 (0.4-2.2)	0.33 (0.09-2.2)	23.5 (14-30)	95.6(39-114)
A4	Fujian	F	3 mos	1.5 yrs	74/51	7.61	40.1	140	2.85	99.0	0.84	320.0	8.71	33.6	1.18 (0.4-2.2)	2.01 (0.09-2.2)	19.2 (14-30)	111.4(30-86)
A5	Tianjin	M	7 yrs	8 yrs	100/70	7.63	49.0	135	2.99	85.7	0.92	410.0	1.18	12.8	0.71 (0.3-1.0)	1.47 (0.04-0.8)	38.0 (25-70)	114.5(89-165)
A6	Anhui	M	2 yrs	7 yrs	75/50	7.47	26.6	137	3.40	96.0	0.83	173.2	1.46	16.5	0.47 (0.3-1.6)	0.14(0.06-1.4)	18.4 (25-70)	171.4(89-165)
A7	Anhui	M	3 mos	3 yrs	72/47	7.55	36.0	134	2.62	96.1	0.94	232.5	6.84	24.2	1.44 (0.4-2.2)	1.68 (0.09-2.2)	24.2 (14-30)	94.4(30-86)
A8	Guizhou	M	3 mos	5 yrs	80/56	7.62	31.9	134	2.54	87.5	0.73	180.6	5.36	50.4	1.56 (0.4-2.2)	0.38 (0.09-2.2)	20.2 (14-30)	107.7(30-86)
A9	Shandong	M	2 mos	1.5 yrs	71/30	7.75	29.3	122	3.30	76.1	0.80	101.2	1.28	46.2	1.49 (0.4-2.2)	0.47 (0.09-2.2)	26.7 (15-33)	81.4(30-86)
A10	Shandong	F	10 mos	4 yrs	98/37	7.57	40.3	137	2.82	85.7	1.07	90.0	1.25	45.1	0.68 (0.4-2.2)	1.33 (0.09-2.2)	19.8 (15-32)	130.0(49-157)
A11	Hunan	M	6 mos	1.5 yrs	95/40	7.90	50.6	133	2.48	85.2	0.89	240.3	14.90	55.7	1.23 (0.4-2.2)	0.89 (0.09-2.2)	9.8 (14-30)	255.2(39-114)
A12	Beijing	F	5 mos	7 yrs	60/43	7.50	33.5	140	3.08	96.4	0.70	262.2	4.56	49.6	1.55 (0.4-2.2)	1.02 (0.09-2.2)	23.1 (14-30)	100.4(39-114)
A13	Jiangxi	M	4 mos	1 yrs	56/30	7.70	35.4	125	2.30	89.1	0.57	600.4	40.12	62.4	2.23 (0.4-2.2)	1.05 (0.09-2.2)	19.5 (14-30)	117.1(39-114)
A14	Liaoning	M	5 mos	1 yrs	78/40	7.64	43.4	119	2.20	82.3	0.90	578.8	38.88	48.4	1.15 (0.4-2.2)	0.38 (0.09-2.2)	20.4 (14-30)	110.2(39-114)
B15	Hunan	M	antenatal	1 yrs	NA	7.53	29.9	129	2.97	89.4	1.02	266.4	19.81	45.2	NA	0.44 (0.09-2.2)	48.8 (37-81)	60.0(17-60)
C16	Jiangsu	F	antenatal	1 yrs	NA	7.56	33.0	128.08	3.09	97.5	0.85	222.8	7.70	56.0	1.44(0.4-2.2)	2.05 (0.09-2.2)	51.0 (37-81)	27.3(17-60)

Figures in parentheses indicate normal ranges of the corresponding age. eGFR was calculated by Schwartz formula. a, age at the first admission; b, present age; mos, months; yrs, years; M, male; F, female; S, serum; U, urine; ALD, aldosterone; PRA, plasma renin activity; NA, not available.

The clinical signs and symptoms from high to low incidence were polydipsia and polyuria (15/16), growth retardation (15/16), constipation (12/16), limb weakness (10/16), convulsions (2/16) and palpitations (2/16).

#### Biochemical features

As can be seen from Table 1, all patients had hypokalemia (average value 2.66±0.50 mmol/L), lower-than-normal serum chloride concentration (average value 88.0±8.9 mmol/L), metabolic alkalosis (average value of CO_2_CP 37.5±7.6 mmol/L), and elevated basal renin activity. 13 patients had elevated aldosterone levels (13/16) and the serum sodium levels of 11 patients were below normal concentration (11/16). 3 showed low serum magnesium, with the urine magnesium/creatinine ratio up to or higher than the upper limit of the normal range. All patients had significantly increased urinary potassium/creatinine ratio. On the other hand, their urinary calcium/creatinine ratios were all within normal range, however, those of two patients (A4 and C16) were close to the upper limit of normal range. No Gitelman-like syndrome (hypokalemia with hypocalciuria (Urine Calcium/Creatinine<0.1 mol/mol)) was found. All patients had normal serum uric acid concentration, except for one patient (C16) with increased value (461 μmol/L, normal range 140-414 μmol/L).

#### Imaging examination

The urinary system ultrasound revealed that both A4 and C16 were complicated with bilateral nephrocalcinosis.

#### Comorbid diseases

Patient A6 was complicated with aplastic anemia, A8 suffered from enamel hypoplasia (Supplementary Figure 1), and B15 was complicated with bilateral sensorineural deafness. Besides, we did not find hearing impairment from the other children.

#### Gene analysis

As shown in Table 2, 15 different mutations of CLCNKB gene were found in 14 cBS patients by high-throughput sequencing and MLPA, including 4 missense mutations (c.887G>A, p.Gly296Asp; c.1052G>T, p.Arg351Leu; c.1294_1295TA>CT, p.Tyr432Leu; c.1333T>G, p.Ser445Ala), 2 nonsense mutations (c.75T>A, p.Cys25^*^; c.88C>T, p.Arg30^*^), 4 small deletions (c.849_851delCTT; c.1000delG; c.1150_1157delCCCCAGCA; c.1657_1664delCACAGCAT), 1 duplication (c.1395dupG), 3 gross deletions (c.(?_-757)_229+?del; c.230-?_^*^410_?del; c.499-?_576+?del) and 1 whole gene deletion mutation (c.(?_-757)_ (^*^410_?)del). Except for the 4 previously reported mutations (c.88C>T; c.887G>A; c.(?_-757)_(^*^410_?)del; c.499-?_576+?del), we have found 11 novel mutations in this study. Both alleles of CLCNKB gene have been detected mutations in each of the 14 cBS probands, the mutation detection rate was 100% accordingly. Among of them, 7 were homozygotes, and 7 were complex heterozygotes. Of note, 3 patients (families) were homozygous deletion of the complete CLCNKB gene, whereas other 3 patients (families) were heterozygous for this deletion, thus the allele frequency of this mutation was 9/28 (32%). And surprisingly, the frequency of large deletions of CLCNKB gene in this cohort of patients was up to 50 percent (14/28). It is noteworthy that patient A11 also carried a heterozygous large deletion of the CLCNKA gene (c.1930-?_^*^412_?del), which is inherited from his father. However, he was still diagnosed as type III BS instead of type IVb BS, since the CLCNKA mutation was detected in a single allele. By whole exome sequencing, we found that patient A8 also carried a novel heterozygous mutation (c.154G>A; p.Ala52Thr) in FAM83H gene which is associated with enamel hypoplasia type 3. However, this mutation was not found in his parents.

**Table 2:** 19 mutations identified in the CLCNKB, CLCNKA, SLC12A1 and BSND genes of the sixteen patients with Bartter syndrome

Patients	gene	Allele 1			Allele 2		
		Nucleotide changes	Amino acid changes	References	Nucleotide changes	Amino acid changes	References
A1	CLCNKB	c.1000delG	p.Val334Phefs^*^15	This study	c.(?_-757)_(^*^410_?)del	-	Simon DB et al [8]
A2	CLCNKB	c.1657_1664delCACAG CAT	p.Ser554Hisfs^*^50	This study	c.1657_1664delCA CAGCAT	p.Ser554Hisfs^*^50	This study
A3	CLCNKB	c.88C>T	p.Arg30^*^	Yu Y et al [9]	c.88C>T	p.Arg30^*^	Yu Y et al [9]
A4	CLCNKB	c.75T>A	p.Cys25^*^	This study	c.849_851delCTT	p.Phe284del	This study
A5	CLCNKB	c.1000delG	p.Val334Phefs^*^15	This study	c.1395dupG	p.Tyr466Valfs^*^56	This study
A6	CLCNKB	c.1294_1295TA>CT	p.Tyr432Leu	This study	c.1333T>G	p.Ser445Ala	This study
A7	CLCNKB	c.1052G>T	p.Arg351Leu	This study	c.499-?_576+?del	-	Colussi G et al [10]
A8, A9, A10	CLCNKB	c.(?_-757)_ (^*^410_?)del	-	Simon DB et al [8]	c.(?_-757)_ (^*^410_?)del	-	Simon DB et al [8]
A11	CLCNKB	c.(?_-757)_229+?del	-	This study	c.(?_-757)_ 229+?del	-	This study
A11	CLCNKA	c.1930-?_^*^412_?del	-	This study			
A12	CLCNKB	c.230-?_^*^410_?del	-	This study	c.230-?_^*^410_?del	-	This study
A13	CLCNKB	c.887G>A	p.Gly296Asp	Vargas-Poussou R et al [11]	c.(?_-757)_ (^*^410_?)del	-	Simon DB et al [8]
A14	CLCNKB	c.1150_1157delCCCCA GCA	P.Gln385Valfs^*^63	This study	c.(?_-757)_ (^*^410_?)del	-	Simon DB et al [8]
B15	BSND	c.22C>T	p.Arg8Trp	Birkenhäger R et al [12]	c.22C>T	p.Arg8Trp	Birkenhäger R et al [12]
C16	SLC12A1	c.1435C>G	p.Leu479Val	This study	1478delG	p.Gly493Alafs^*^53	This study

Mutation naming and description rules refer to the latest guideline published by Human Genome Variation Society (http://www.hgvs.org/mutnomen/recs.html).

Regarding these two aBS patients, patient B15 was identified to harbor a previously reported homozygous missense mutation of BSND (c.22C>T; p.Arg8Trp), whereas patient C16 was revealed to carry two novel mutations (complex heterozygous) of SLC12A1 (c.1435C>G, p.Leu479Val; c.1478delG, p.Gly493Alafs^*^53).

All of the above mentioned 13 novel mutations were not found in the 200 healthy controls.

Grantham-Matrix scoring system and the three types of *in silico* software (SIFT, PolyPhen-2 and MutationTaster) were employed to predict the pathogenicity of the novel missense mutations, including three mutations of CLCNKB (c.1052G>T, p.Arg351Leu; c.1294_1295TA>CT, p.Tyr432Leu; c.1333T>G, p.Ser445Ala) and one missense variant of SLC12A1 (c.1435C>G, p.Leu479Val) (Supplementary Table 1 & 2). It turned out that both Grantham-Matrix scoring system and the three types of online software predicted p.Arg351Leu as a deleterious mutation with high probability. p.Tyr432Leu, p.Ser445Ala and p.Leu479Val were predicted to be harmful with moderate probability by Grantham-Matrix scoring system. However, p.Tyr432Leu and p.Ser445Ala were predicted to be benign by the three online programs, with the exception that p.Leu479Val was harmful. Sequence alignment demonstrated that the sites of these missense mutations (p.R351, p.Y432 and p.S445) were conserved in 7, 8 and 5 species of clc-kb homologous proteins respectively, while p.L479 was highly conserved in all of these 8 species of NKCC2. Additionally, all of the four novel missense mutations, including these alterations of p.Arg351Leu, p.Tyr432Leu and p.Ser445Ala of CLCNKB gene and the variant of p.Leu479Val of SLC12A1 gene, have never been reported to the databases of 1000G, ExAC and HGMD before.

Except for the above-mentioned missense mutation, we have also found other 6 different nonsynonymous single nucleotide changes in CLCNKB gene (c.1291G>A, p.Val431Leu; c.1327A>G, p.Thr443Ala; c.1336T>G, p.Phe446Val; c.1340T>C, p.Ile447Thr; c.1360G>A, p.Ala454Thr; c.1369A>G, p.Ile457Val) (Supplementary Table 1 & 2). Of note, in the HGMD database, p.Ile447Thr has been reported as heterozygous state in a patient with Gitelman-like syndrome [11].

### Treatment and follow-up

**Figure 1:**
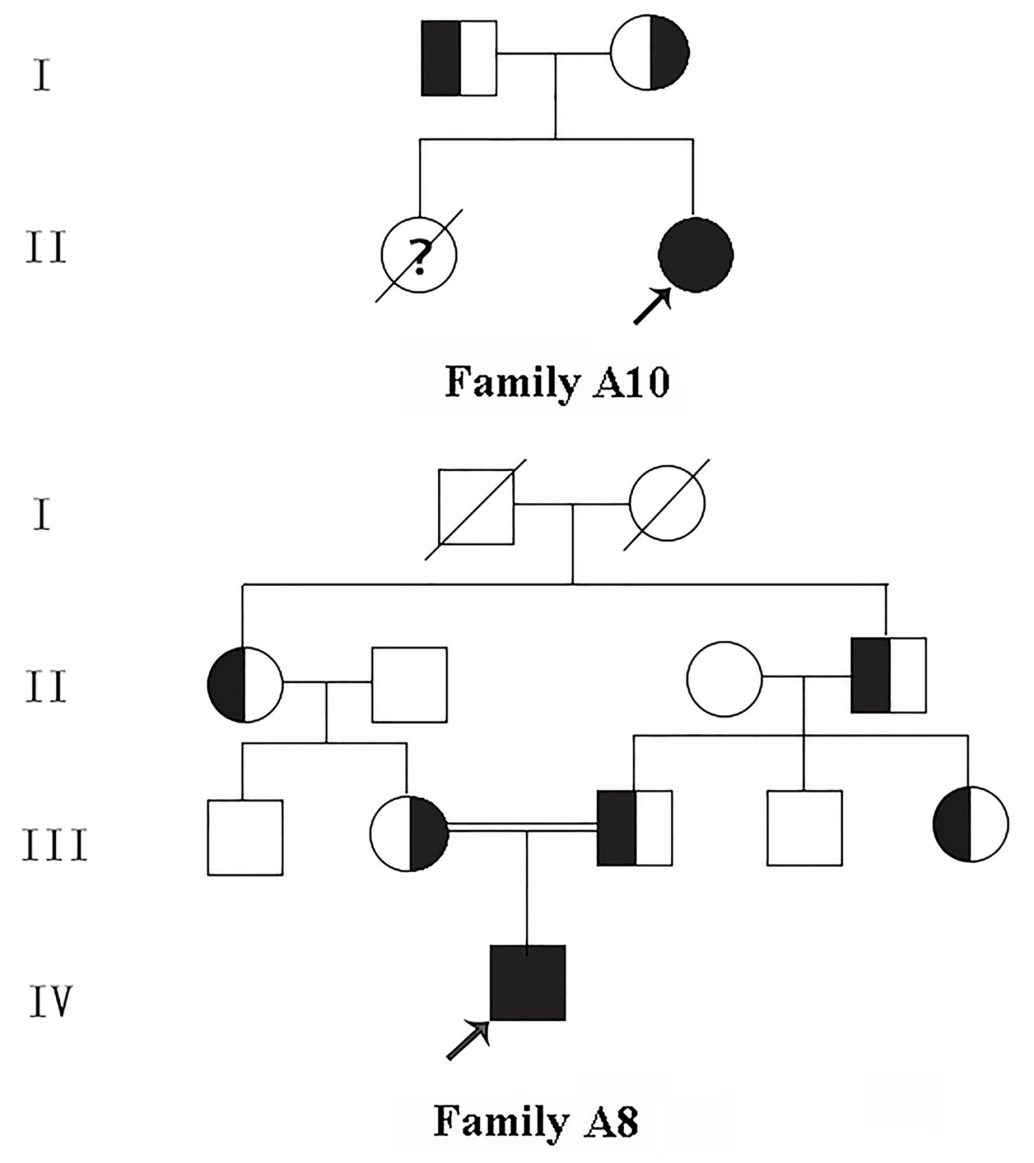
Pedigree of the kindred A8 and A10 □, Male; ○, Female; ■, Male patient; ●, Female patient; ↗, Proband; ?, Suspect.

The treatment regimen was shown in Supplementary Table 3, and the adjustment of therapeutic program was based on the degree of recovery from growth retardation and electrolyte disturbance. The growth curves of these patients (height and weight) and the time of medication intervention were shown in Figure 1.

In this cohort of patients, the height and/or body weight of the majority (15/16) were at least 1SD lower than the mean values, except for patient A5 who did not manifest growth retardation before 5.5 years old. Of note, the retardation of body weight was usually more severe than height in our patients.

Among these 15 patients with growth retardation, 11 achieved obvious recovery of growth rate with an indomethacin-based combination therapy. In the other 4 patients without significant recovery of growth and development (4/15), patient A1, A14 and C16 were only treated by potassium chloride (1/15) or combined with spironolactone (2/15) instead of indomethacin. Patient B15 had received a treatment regimen of both indomethacin and potassium chloride, but the improvement of growth speed was not evident. Besides, there was no statistically significant difference (3.3 ± 0.35 vs 3.5 ± 0.44, p = 0.22) in the levels of serum potassium between the patients treated with indomethacin (n = 10, excluding 2 patients with renal insufficiency) and those without indomethacin (n = 4).

In recent regular reviews, patient A10 and B15 were detected with apparent declining of glomerular filtration rate, which were 47.8 mL·min^-1^·1.73m^2-1^ (reference: 89-165 mL·min^-1^·1.73m^2-1^) and 25.5 mL·min^-1^·1.73m^2-1^ (reference value: 49-157 mL·min^-1^·1.73m^2-1^), respectively.

## DISCUSSION

By gene analysis, 14 patients were diagnosed with BS type III (14/16), the other 2 cases were respectively detected with BS type I (1/16) and BS type 4a (1/16), indicating that among the various types of BS, the prevalence of type III was likely the highest in Chinese population. However, the prevalence of antenatal forms of BS might be underestimated because of the high rate of premature death. Besides that, our conclusion should be verified in a larger cohort of Chinese patients.

Among those 14 patients with type III BS, 15 mutations of CLCNKB gene were identified, including 11 novel ones which expanded the number of mutations of CLCNKB gene in HGMD from 96 mutations to 107. Obviously, except for 3 missense and 1 in-frame deletion mutations, all the other novel ones were severe pathogenic mutations which either led to a failure to initiate the process of translation, or resulted in the production of seriously truncated non-functional protein, or caused a reduction in mRNA levels due to the nonsense-mediated mRNA decay (NMD). While the mutation c.849_851delCTT resulted in a deletion of the 284^th^ phenylalanine, which is located in the α helix of the 10^th^ J trans-membrane region, other 3 previously described missense mutations (p.Gly296Asp, p.Ser297Arg and p.Gln303Pro) are also localized in this trans-membrane helix [11, 13, 14]. Besides, the 284^th^ phenylalanine lied in a highly conserved domain, its deletion may represents pathogenicity, but still requiring *in vitro* functional studies to confirm. As for the 3 novel missense mutations, p.Arg351Leu is located between the trans-membrane helices K and L, and 2 other forms of mutations in the same site (p.Arg351Pro, p.Arg351Trp) have been confirmed to be pathogenic [9, 15]. The mutation p.Tyr432Leu is located in α-helix N of ClC-Kb, which is involved in the selectivity filter. It probably be morbigenous because another variant p.Tyr432His in the same site from a published case with BS had been validated to be pathogenic by functional expression *in vitro* [8, 16], meanwhile the extreme rarity and high conservation of this variant also support this presumption, although the inconsistent predictive results from *in silico* software and Grantham-Matrix scoring system. Likewise, the mutation p.Ser445Ala from the same patient with p.Tyr432Leu but a different allele, which is localized at the junction of α-helix N and the following extracellular region, might also be damaging, however further research is needed to confirm its deleteriousness.

In this study, we found that the deletion of complete CLCNKB gene was the most common mutation in Chinese patients with cBS, this finding was consistent with most studies based on Caucasian [2, 8, 13]. In addition, the frequency of large deletions was up to 14/28 (50%), suggesting that in Chinese population, technical methods of genetic diagnosis for BS should be good in identification of large deletions, such as a combination of second-generation sequencing and MLPA, and this may be exactly the reason why the mutation detection rate in this study was up to 100%.

Two patients with aBS were confirmed to be type I and type IVa BS by gene analysis, respectively. Patient C16 carried two novel mutations (complex heterozygous) of the SLC12A1 gene. One missense mutation c.1435C>G (p.Leu479Val) is located in the extracellular segment between the 7^th^ and 8^th^ trans-membrane helices of NKCC2, and the adjacent p.Gly478Ala has been identified to be a causal mutation in aBS [17]. The other one was a small deletion c.1478delG, which led to a frame shift from the 493^rd^ glycine, and to the premature termination at codon 545 (p.Gly493Alafs^*^53), leading to a truncated protein. Patient B15 was identified to harbor a homozygous missense mutation c.22C> T (p.Arg8Trp) of BSND gene, which has been previously reported and was associated with a similar phenotype such as deafness and renal failure [12].

It is noteworthy that two patients in this study had also been detected to carry a second gene mutation. Patient A11 was found to harbor a novel heterozygous deletion of the last two exons of CLCNKA gene. While patient A8 carried a novel missense mutation (c.154G>A; p.Ala52Thr) of FAM83H gene (the related gene of enamel hypoplasia type III, autosomal dominant inheritance), which was predicted to be moderately pathogenic by *in silico* analysis of SIFT, Polyphen 2 and MutationTaster. However, whether the enamel defect in this child was the result of the mutation in FAM83H gene, or was the rare manifestation of BS, still requires more research to determine. Consequently, with the widespread use of high-throughput sequencing technology, more and more patients are going to be identified that their complex clinical phenotype may be attributed to double or triple mutant genes, and this will deepen our understanding of complicated disorders.

Concerning biochemical findings, unlike previous studies [2, 13, 18–20], no patient with Gitelman-like syndrome was found in our patients with type III BS, this might be explained by the following reasons. Firstly, our research subjects were made up of infants and children, however, Gitelman-like syndrome was mainly found in adult-onset patients [2]. Secondly, hypocalciuria may be associated with particular mutations in the CLCNKB gene such as the most prevalent variant c.1830G>A in Japanese and Korean patients [18–19], whereas such mutations were not found in this cohort. Thirdly, there was a trend from BS phenotype to Gitelman-like phenotype conversion over time, and Gitelman-like phenotype may occur among our patients in the future follow-up. Lastly, the high frequency of gross deletions and entire gene deletion may imply more severe disease and with greater urinary salt wasting and hypercalciuria in our patients. Additionally, the prevalence of hyperuricemia or gout in patients with BS was reported to be nearly 50% in the European-American population [21], only one case with hyperuricemia (1/16) was found in this cohort of patients however. This discrepancy might be related to the difference of race or eating habits.

Consistent with some previous studies, it was also difficult to determine the relationship between genotype and phenotype in patients with BS type III in this study [20]. For example, patient A5 carried two severe frameshift mutations (c.1000delG & c.1395dupG, complex heterozygous), but his onset of BS was significantly later than other patients, and his growth development was not complicated. The reason that the correlations of genotype and phenotype in BS type III are difficult to determine may be related to the modification of other genes that involve salt intake and/or reabsorption (e.g. CLCNKA) or various dietary habits (e.g. daily salt intake). However, a rather elaborate analysis based on larger population and combined with functional studies of associated mutations might be required to determine the phenotype/genotype correlations [2].

Except for one with type III BS who had normal growth development, the other 15 patients in this study suffered from growth retardation. Fortunately, 11 patients obtained significant improvement of delayed growth after acceptation of the indomethacin-based combination therapy. Among the four patients with poor response to treatment, three were not treated with indomethacin and the other one suffered from type 4a BS. However, there was no significant difference in serum levels of potassium and chlorine between patients treated with indomethacin or not. Hence, at least partly, the effect of indomethacin on restoration of the delayed growth was likely independent of blood electrolytes levels. Additionally, our study demonstrated that type 4a BS had poor response to indomethacin once again, but lack effective treatment presently [22]. Until now, in this cohort of patients, we had not found serious side effect such as colon perforation that had been described by other investigators [23]. The eGFR of all the 16 patients were within normal range at the initial visit, however, renal insufficiency began to emerge in patient A10 and B15 at the last visit. This result may be consistent with previous finding that chronic renal failure is not a rare event, associated with all genotypes and not always associated with nephrocalcinosis [2, 24].

Of note, our 16 patients scattered in various areas of China, and the clinicians of local hospitals lacked the knowledge and experience of BS treatment. For this reason, these patients were short of normative therapy and guidance. And one of the shortcomings of this investigation was that the period of follow-up was not long enough, the electrolyte levels on treatment had not regained normal range in half of patients, and the growth rates might not achieve the ideal state, and the most appropriate treatment project or therapeutic dose still required adjustment over time.

In summary, we have found 19 mutations in 4 BS genes, including 15 mutations in CLCNKB gene, two in SLC12A1 gene, one in BSND gene and one in CLCNKA gene. Among them, 14 ones were novel. These findings further expanded the mutation spectrum of BS. We have also found that the deletion of complete CLCNKB gene was the most common mutation in Chinese population, and the allele frequency of large deletion was as high as 50%, however, no missense or nonsense hot spot of mutation was identified. This investigation not only revealed the mutation characteristics of Chinese patients with BS, but also provided valuable reference data for genetic counseling and diagnosis.

## MATERIALS AND METHODS

### Research subjects

Criteria for the diagnosis of BS included polydipsia and polyuria, hypochloremic metabolic alkalosis, evidence of renal salt wasting, activation of the renin-angiotensin-aldosterone system associated with normal to low blood pressure. Secondary BS or pseudo-BS due to long-term use of laxatives, diuretics and cystic fibrosis was excluded. Gitelman syndrome confirmed by gene diagnosis was also not included.

**Figure 2:**
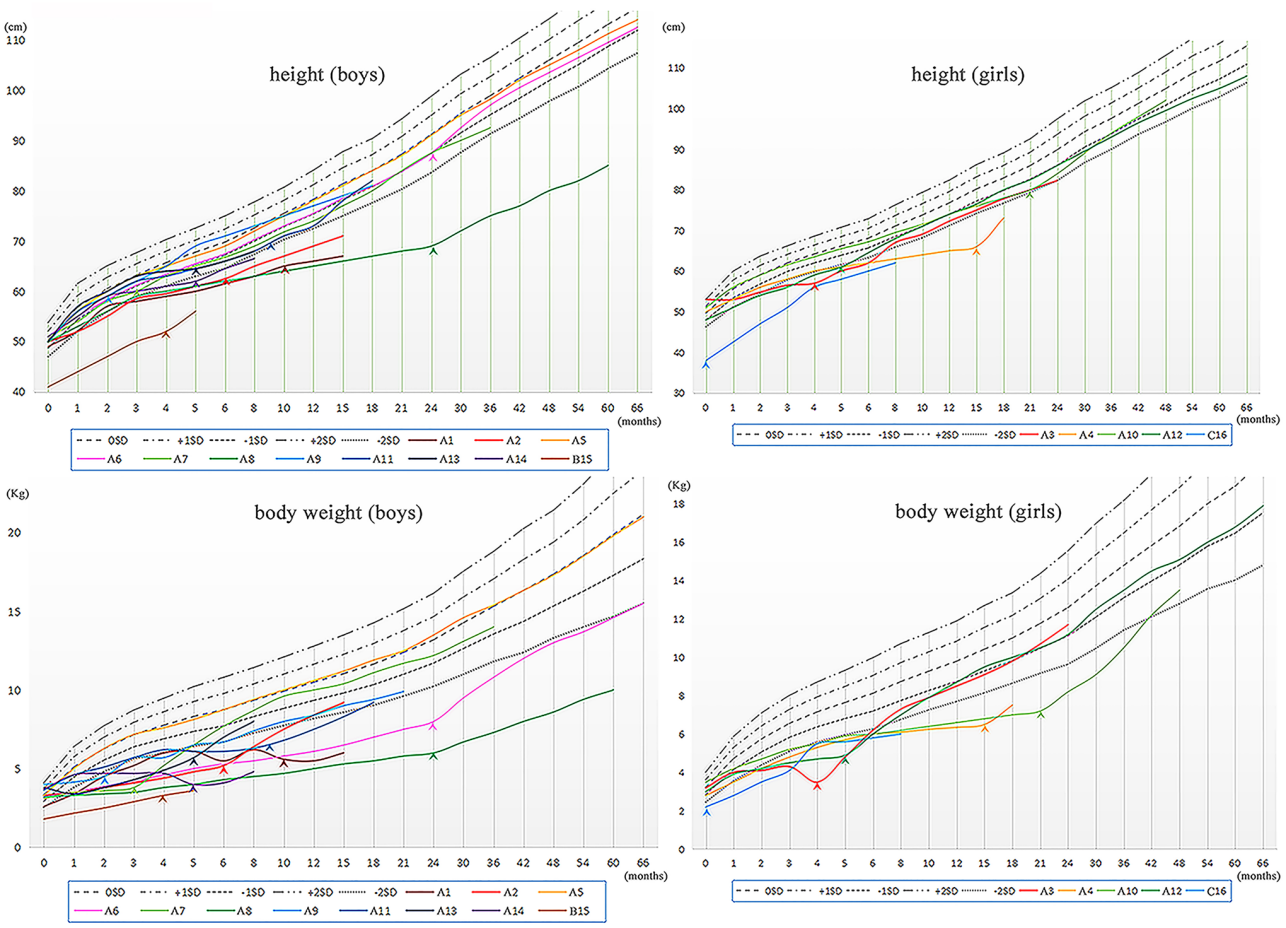
The Growth Curves of the sixteen patients with Bartter syndrome Arrow heads, start of treatment; SD, standard deviation.

This study recruited 16 patients from 16 unrelated families who had been hospitalized in our nephrology department of Qingdao University Affiliated Hospital from June 2010 to January 2017, including 2 cases of aBS and 14 cases of cBS, of which 11 were male, 4 were female. Their average age was 2.9±2.5 years old. One family was from Hui Chinese population, the other 15 families were from Han Chinese population, and the parents of Patient A8 were consanguineous (Pedigree of kindred A8 & A10 see Figure 2). 200 healthy subjects from different families were selected as control to assess new mutations identified in this study. This study was approved by the Ethics Committee of Affiliated Hospital of Qingdao University, and all participants signed informed consent.

### Mutation analysis

#### Genomic DNA extraction

Genomic DNA was extracted from peripheral blood of these probands and their family numbers by GenElute blood genomic DNA kit (Sigma, NA2010).

#### High-throughput sequencing

High-throughput sequencing was used to analyze the exon regions and flanking intronic regions of 7 genes associated with BS and Gitelman syndrome (on patient A4, A5, A6, A7, A9, A10, A13 and C16), or the 136 genes related to hereditary nephropathy (on patient A1, A2, A11, A12 and B15), or the genes of more than 4,000 kinds of human genetic diseases (on patient A3, A8 and A14). Reads that passed were then aligned to the human reference genome (UCSC hg19) using the Burrows-Wheeler Aligner (University of California, Santa Cruz, CA, USA). The variant call file (VCF) containing these variants was annotated with Variant Effect Predictor v83 and the dbNSFP (Database for Nonsynonymous SNPs’ Functional Predictions) v3.1.

After the selection process, we use the online software like SIFT, PolyPhen-2 and MutationTaster to predict the pathogenicity of putative missense mutations. On the basis of matrix algorithm described by Grantham and Abkevich et al [6–7], further score system evaluating the deleteriousness of missense mutation was used in this study. Then, we performed sequence alignment on 8 species for clc-kb (the production of CLCNKB) and NKCC2 (the production of SLC12A1), respectively, through Vector NTI Advance 11.5-Align. Those 8 species for clc-kb were as follows: mus musculus (NP_062675), macaca mulatta (XP_014975670), rattus norvegicus (NP_775126), chelonia mudas (XP_007053197), gallus gallus (XP_015152516), pan troglodytes (XP_016810262), xenopus tropicalis (XP_017945789) and human (NP_000076); and those for NKCC2: mus musculus (NP_899197), oryctolagus cuniculus (NP_001164442), ailuropoda melanoleuca (XP_002917595), canis lupus familiaris (XP_005638445), macaca mulatta (XP_014997658), danio rerio (XP_009291678), rattus norvegicus (NP_001257546) and human(NP_000329).

#### MLPA verification

Multiplex ligation-dependent probe amplification (MLPA) was performed to verify large deletions and duplications in CLCNKB gene. MLPA analysis was done according to the protocol supplied by the manufacturer of the SALAS-MLPA Kit P266 (MRC-Hollad, Amsterdam, The Netherlands) which containing 14 probes specific for CLCNKB gene (exon 1, 2, 3, 5, 6, 8, 10, 11, 13, 14, 15, 17, 18 & 19) and 2 probes for CLCNKA gene. In addition, 11 reference probes located on various chromosomes was used as intrasample normalization.

#### Sanger sequencing verification

The potential candidate variant was validated by Sanger sequencing in patients and their family members. The suspected candidate mutation sites and its flanking regions were amplified by PCR and underwent direct Sanger Sequencing using an ABI Prism 3700 DNA Analyzer (Applied Biosystems, Calif., USA).

### Statistical analysis

The biochemical data were expressed as mean±SD. The Student's unpaired *t*-test was used to compare the differences between the patients treated with indomethacin and those treated without indomethacin. P<0.05 was considered statistically significant.

## SUPPLEMENTARY MATERIALS FIGURES AND TABLES



## References

[1] Matsunoshita N, Nozu K, Shono A (2016). Differential diagnosis of Bartter syndrome, Gitelman syndrome, and pseudo-Bartter/Gitelman syndrome based on clinical characteristics. Genet Med.

[2] Seys E, Andrini O, Keck M Clinical and Genetic Spectrum of Bartter Syndrome Type 3. J Am Soc Nephrol.

[3] Seyberth HW (2008). An improved terminology and classification of Bartter-like syndromes. Nat Clin Pract Nephrol.

[4] Laghmani K, Beck BB, Yang SS, Seaayfan E, Wenzel A, Reusch B, Vitzthum H, Priem D, Demaretz S, Bergmann K, Duin LK, Göbel H, Mache C (2016). Polyhydramnios, Transient Antenatal Bartter's Syndrome, and MAGED2 Mutations. N Engl J Med.

[5] Dong Y, Ji G, Feng QW, Zeng XT, Jiang GR (2010). A novel splicing mutation in CLCNKB in a Chinese patient with Bartter syndrome type III. Chin Med J (Engl).

[6] Grantham R (1974). Amino acid difference formula to help explain protein evolution. Science.

[7] Abkevich V, Zharkikh A, Deffenbaugh AM (2004). Analysis of missense variation in human BRCA1 in the context of interspecific sequence variation. J Med Genet.

[8] Simon DB, Bindra RS, Mansfield TA (1997). Mutations in the chloride channel gene, CLCNKB, cause Bartter's syndrome type III. Nat Genet.

[9] Yu Y, Xu C, Pan X (2009). Identification and functional analysis of novel mutations of the CLCNKB gene in Chinese patients with classic Bartter syndrome. Clin Genet.

[10] Colussi G, De Ferrari ME, Tedeschi S (2002). Bartter syndrome type 3: an unusual cause of nephrolithiasis. Nephrol Dial Transplant.

[11] Vargas-Poussou R, Dahan K, Kahila D (2011). Spectrum of mutations in Gitelman syndrome. J Am Soc Nephrol.

[12] Birkenhäger R, Otto E, Schürmann MJ (2001). Mutation of BSND causes Bartter syndrome with sensorineural deafness and kidney failure. Nat Genet.

[13] Konrad M, Vollmer M, Lemmink HH (2000). Mutations in the chloride channel gene CLCNKB as a cause of classic Bartter syndrome. J Am Soc Nephrol.

[14] Urbanová M, Reiterová J, Stěkrová J, Lněnička P, Ryšavá R (2011). DNA analysis of renal electrolyte transporter genes among patients suffering from Bartter and Gitelman syndromes: summary of mutation screening. Folia Biol (Praha).

[15] Keck M, Andrini O, Lahuna O (2013). Novel CLCNKB mutations causing Bartter syndrome affect channel surface expression. Hum Mutat.

[16] Waldegger S, Jentsch TJ (2000). Functional and structural analysis of ClC-K chloride channels involved in renal disease. J Biol Chem.

[17] Vargas-Poussou R, Feldmann D, Vollmer M (1998). Novel molecular variants of the Na-K-2Cl cotransporter gene are responsible for antenatal Bartter syndrome. Am J Hum Genet.

[18] Nozu K, Iijima K, Kanda K, Nakanishi K, Yoshikawa N, Satomura K, Kaito H, Hashimura Y, Ninchoji T, Komatsu H, Kamei K, Miyashita R, Kugo M (2010). The pharmacological characteristics of molecular-based inherited salt-losing tubulopathies. J Clin Endocrinol Metab.

[19] Lee BH, Cho HY, Lee H, Han KH, Kang HG, Ha IS, Lee JH, Park YS, Shin JI, Lee DY, Kim SY, Choi Y, Cheong HI (2012). Genetic basis of Bartter syndrome in Korea. Nephrol Dial Transplant.

[20] García Castaño A, Pérez de Nanclares G, Madariaga L (2015). Poor phenotype-genotype association in a large series of patients with Type III Bartter syndrome. PLoS One.

[21] Meyer WJ, Gill JR, Bartter FC (1975). Gout as a complication of Bartter's syndrome. A possible role for alkalosis in the decreased clearance of uric acid. Ann Intern Med.

[22] Jeck N, Reinalter SC, Henne T (2001). Hypokalemic salt-losing tubulopathy with chronic renal failure and sensorineural deafness. Pediatrics.

[23] Ataoglu E, Civilibal M, Ozkul AA (2009). Indomethacin-induced colon perforation in Bartter's syndrome. Indian J Pediatr.

[24] Brochard K, Boyer O, Blanchard A (2009). Phenotype-genotype correlation in antenatal and neonatal variants of Bartter syndrome. Nephrol Dial Transplant.

