# Supplementary material for: Mutation spectrum of Chinese patients with Bartter syndrome
Source: Oncotarget. 2017 Sep 27;8(60):101614–22. doi: 10.18632/oncotarget.21355 (PMC5731900; doi:10.18632/oncotarget.21355)
Supplement: Supplementary file 1 [file oncotarget-08-101614-s001.pdf]

Mutation spectrum of Chinese patients with Bartter syndrome

SUPPLEMENTARY MATERIALS

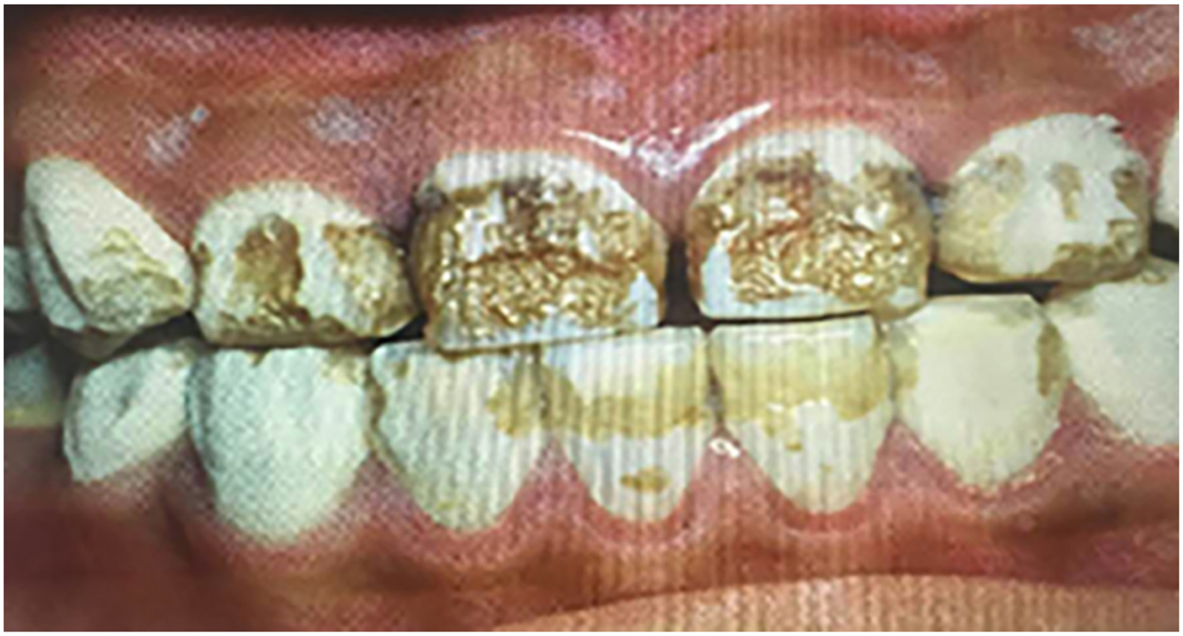

Supplementary Figure 1: The picture of enamel hypoplasia of patient A8. The crowns of some teeth were chipped away and the underlying yellow dentin was exposed in this boy.

Supplementary Table 1: Pathogenicity scoring of the ten novel nucleotides variants of CLCNKB and SLC12A1 in this study

No.	Gene	Gene mutation	Protein mutation	Grantham Matrix Scoring	MSA Scoring	Total Score
1	CLCNKB	c.1052G>T	p.Arg351Leu	102(4)	7/8(4)	8
2	CLCNKB	c.1291G>A	p.Val431Leu	32(1)	5/8(3)	4
3	CLCNKB	c.1294_1295TA>CT	p.Tyr432Leu	61(2)	8/8(5)	7
4	CLCNKB	c.1327A>G	p.Thr443Ala	58(1)	3/8(1)	2
5	CLCNKB	c.1333T>G	p.Ser445Ala	99(4)	5/8(3)	7
6	CLCNKB	c.1336T>G	p.Phe446Val	50(1)	4/8(3)	4
7	CLCNKB	c.1340T>C	p.Ile447Thr	89(3)	4/8(3)	6
8	CLCNKB	c.1360G>A	p.Ala454Thr	58(1)	6/8(4)	5
9	CLCNKB	c.1369A>G	p.Ile457Val	29(1)	4/8(3)	4
10	SLC12A1	c.1435C>G	p.Leu479Val	32(1)	8/8(5)	6

Grantham Matrix scoring: < 60.0 = 1 point, 60.0– 78.3 = 2 points, 78.4–93.4 = 3 points, > 93.4 = 4 points, any substitution of cysteine = 5 points. MSA scoring: conservative in all eight of species = 5 points, 6-7 species = 4 points, 4-5 species = 3 points, 1-3 species = 1 point. Total score: ≥ 8 high pathogenicity, 6-8 moderate pathogenicity, ≤5 probably nonpathogenic (like polymorphism). MSA: multiple sequence alignment.

Supplementary Table 2: Pathogenicity prediction results of ten novel nucleotides variants of CLCNKB and SLC12A1 by SIFT, PolyPhen-2 and Mutation Taster

No.	Gene	Nucleotide changes	Amino-acid change	SIFT	PolyPhen-2	Mutation Taster
1	CLCNKB	c.1052G>T	p.Arg351Leu	Damaging	Probably Damaging	Disease causing
2	CLCNKB	c.1291G>A	p.Val431Leu	Tolerated	Benign	Polymorphism
3	CLCNKB	c.1294_1295TA>CT	p.Tyr432Leu	Tolerated	Benign	Polymorphism
4	CLCNKB	c.1327A>G	p.Thr443Ala	Tolerated	Benign	Polymorphism
5	CLCNKB	c.1333T>G	p.Ser445Ala	Tolerated	Benign	Polymorphism
6	CLCNKB	c.1336T>G	p.Phe446Val	Tolerated	Benign	Polymorphism
7	CLCNKB	c.1340T>C	p.Ile447Thr	Tolerated	Benign	Polymorphism
8	CLCNKB	c.1360G>A	p.Ala454Thr	Tolerated	Benign	Disease causing
9	CLCNKB	c.1369A>G	p.Ile457Val	Tolerated	Benign	Polymorphism
10	SLC12A1	c.1435C>G	p.Leu479Val	Damaging	Probably Damaging	Disease causing

Supplementary Table 3: The treatment regimen and the after-therapy laboratory results of the sixteen patients with Bartter syndrome

Patient	Indomethacin ($mg \cdot Kg^{-1} \cdot d^{-1}$)	Potassium chloride ($g \cdot Kg^{-1} \cdot d^{-1}$)	Spironolactone ($mg \cdot Kg^{-1} \cdot d^{-1}$)	Serum potassium (mmol/L)	Serum Chlorine (mmol/L)	CO_2CP (mmol/L)	eGFR($mL \cdot min^{-1} \cdot 1.73\ m^{2-1}$)
A1	0.00	1.17	1.33	3.76	95.3	26	239.2
A2	2.01	1.05	0.00	3.52	94.6	25	158.6
A3	1.50	0.60	0.80	2.81	90.4	29	192.3
A4	1.25	0.38	0.00	3.30	97.2	22	93.2
A5	0.00	0.51	1.15	3.05	92.0	34	138.5
A6	0.46	0.22	1.43	3.40	96.2	27	197.7
A7	0.89	0.57	1.40	3.52	99.1	25	147.3
A8	1.75	0.40	2.00	3.10	89.3	32	128.9
A9	2.50	0.60	3.70	4.01	102.5	25	108.6
A10	2.71	0.40	0.65	3.23	92.7	30	47.8
A11	1.36	0.33	0.00	3.59	101.6	26	157.5
A12	0.53	0.46	2.50	3.62	93.0	33	136.7
A13	2.08	0.28	2.80	4.41	98.5	26	129.4
A14	0.00	0.38	3.13	3.47	91.0	23	117.8
B15	1.95	1.09	0.00	4.69	80.3	20	25.5
C16	0.00	0.19	0.00	3.01	99.0	27	181.2

eGFR was calculated by Schwartz formula.
